# Using Non-linear Dynamics of EEG Signals to Classify Primary Hand Movement Intent Under Opposite Hand Movement

**DOI:** 10.3389/fnbot.2022.845127

**Published:** 2022-04-28

**Authors:** Jiarong Wang, Luzheng Bi, Weijie Fei

**Affiliations:** School of Mechanical Engineering, Beijing Institute of Technology, Beijing, China

**Keywords:** EEG, hand movement decoding, human augmentation, human factors, human-machine interaction

## Abstract

Decoding human hand movement from electroencephalograms (EEG) signals is essential for developing an active human augmentation system. Although existing studies have contributed much to decoding single-hand movement direction from EEG signals, decoding primary hand movement direction under the opposite hand movement condition remains open. In this paper, we investigated the neural signatures of the primary hand movement direction from EEG signals under the opposite hand movement and developed a novel decoding method based on non-linear dynamics parameters of movement-related cortical potentials (MRCPs). Experimental results showed significant differences in MRCPs between hand movement directions under an opposite hand movement. Furthermore, the proposed method performed well with an average binary decoding accuracy of 89.48 ± 5.92% under the condition of the opposite hand movement. This study may lay a foundation for the future development of EEG-based human augmentation systems for upper limbs impaired patients and healthy people and open a new avenue to decode other hand movement parameters (e.g., velocity and position) from EEG signals.

## Introduction

Human augmentation refers to using assistive devices and technologies to help people overstep human motor, perception, and cognition limitations. The applications of human augmentation have shown diversity, including but not limited to prostheses (Kvansakul et al., 2020), exoskeleton (Chen et al., 2017; Yandell et al., 2019), and augmented reality (Kansaku et al., 2010; Chen et al., 2021). For human augmentation systems, providing active assistance instead of passive assistance according to human intention is of high value. Fusing human intention into augmentation technology makes it possible to establish a more intelligent, flexible, and user-friendly system.

Brain-computer interfaces (BCIs) have been the essential tool to detect human intention with the development of neuroscience. BCIs could translate human intention from neural signals directly. Among various brain signal recording methods, electroencephalogram (EEG) is more practical for human augmentation because it is non-invasive, cheap, and convenient to use. Over the past decades, numerous studies have been focused on using EEG signals to decode human intention and develop a body augmentation system, e.g., P300 speller (Farwell and Donchin, 1988), steady-state visually evoked potential-based BCI systems (Gao et al., 2021), e.g., exoskeleton control (Kwak et al., 2015) and motor rehabilitation (Zhao et al., 2016), motor imagery (MI)-based mobile wheelchair (He et al., 2016; Zhang et al., 2016), movement-related cortical potential (MRCP)-based robotic arm control (Schwarz et al., 2020a,b). Compared with evoked potentials-based BCIs (Yin et al., 2015a,b) and MI-based BCIs (Pei et al., 2022), MRCP-based BCIs do not rely on external evoked stimuli (such as P300) and repetitive imagination (such as MI). It can decode human intention from natural movement execution and provide a more realistic application scene for human augmentation.

MRCP-based hand (or arm) movement intention decoding is an important branch of MRCP-based movement intention decoding. Existing studies on upper limb movement intention decoding include movement parameters decoding (e.g., direction (Robinson et al., 2013; Chouhan et al., 2018), position (Hammon et al., 2008; Sosnik and Zheng, 2021), velocity (Robinson et al., 2013; Ubeda et al., 2017; Korik et al., 2018), acceleration (Bradberry et al., 2009), handgrip force (Haddix et al., 2021) and movement type recognition (Ofner et al., 2019). Reviewing existing studies about upper limb movement decoding, we find that most existing studies are concentrated on single hand (or arm) movement decoding. However, for the practical application of human augmentation, both-hand movement is common. Considering this issue, in 2020, Schwarz et al. (2020) first used the low-frequency EEG features to discriminate unimanual and bimanual daily reach-and-grasp movement types and achieved a multi-class classification accuracy of 38.6% for a combination of one rest and six movement types. Furthermore, to put the single-hand and both-hand movement intention decoding from EEG signals into an active human augmentation system, in 2020, we investigated the neural signatures and classification of single-hand and both-hand movement directions, and the 6-class classification achieved a peak accuracy of 70.29% (Wang et al., 2021).

It should be noted that both studies by Schwarz et al. (2020) and Wang et al. (2021) are focused on the discrimination of single-hand and both-hand movement. However, it is not enough to discriminate single-hand movement from both-hand movement. In many both-hand movement cases, we value the primary hand movement (e.g., the movement direction, velocity, or trajectory of single right hand) instead of whether we move one hand or both hands. Thus, it is necessary to decode the primary hand movement under the opposite hand movement condition. To solve the problem, in this study, we stride the first step by investigating the decoding of the movement direction of the primary hand (i.e., right hand in this paper) from EEG signals recorded during the opposite hand (i.e., left hand in this paper) movement. Notably, in this paper, we define the movement condition with the opposite hand movement as “W-OHM.”

The contribution of this paper is that it is the first work to investigate the neural signatures and decoding of primary hand movement direction from EEG signals under the opposite hand movement and propose a novel decoding method based on non-linear dynamics parameters of MRCPs. This work not only can lay a foundation for the future development of BCI-based human augmentation systems for upper limbs impaired patients and healthy people, but it also may open a new avenue to decode other hand movement parameters (e.g., velocity and position) from EEG signals.

The remainder of the paper is structured as follows: section Methods introduces the methods. Section Results shows the results. Section Discussion and Conclusion presents the discussion, limitations of our work, and future work.

## Methods

### Experimental Paradigm and Procedure

**Figure 1:**
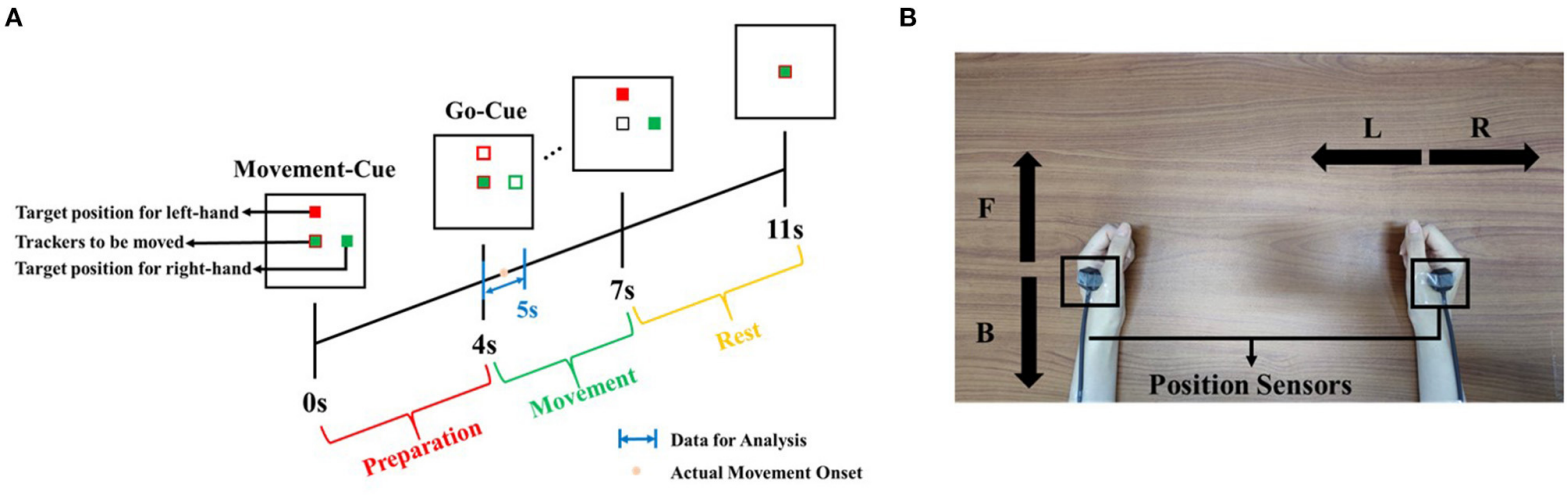
Experimental protocol. **(A)** Timeline of experiment setup. Note that time 4 s refers to the Go-Cue, and time 4.5 s is the actual movement onset. **(B)** Illustration of both hand movement directions. Note that “F” and “B” refer to the movement of left-hand in the forward or backward direction, respectively, and “R” and “L” refer to the movement of right-hand in the right and left direction, respectively.

We recruited and measured 14 participants (one female), aged between 22 and 27 years. They reported having normal vision and no brain diseases. According to the Hand-Dominance-Test, they were all confirmed to be right-handed (Bryden, 1977). The study adhered to the principles of the 2013 Declaration of Helsinki. The research was approved by the local research ethics committee. All data were recorded at the IHMS Lab of the School of Mechanical Engineering, Beijing Institute of Technology, China. Subjects were seated on a chair in a room free of noise and electromagnetic interference. In front of them, there was a monitor for experimental instructions. Figure 1 shows the experimental protocol.

Considering that all subjects were right-handed, we regarded the right-hand movement as the primary movement to be decoded and the left-hand movement as the opposite hand movement. For the primary movement task, all subjects were required to move their right hands in right or left directions. We preliminarily set the opposite hand movement in the vertical directions rather than horizontal directions. All subjects were asked to move their left hands in forward and backward directions. The movement of both hands was restricted in the horizontal plane parallel to the desktop. We defined the movement of right-hand in the right or left direction as “*R*” or “*L*” and the movement of left-hand in the forward or backward direction as “*F*” or “*B*.” As shown in Figure 1A, on the monitor, two solid blocks colored as red and green correspond to the movement cue of left and right hands, respectively. When one trial was initiated, the red block would randomly appear in the *F* or *B* directions, and the green block would randomly appear in the *L* or *R* directions. That means, after 0 s (movement-cue onset), subjects were indicated for the movement directions and prepared for the movement. At the fourth second, both blocks changed from the solid into hollow, which were regarded as go-cue. Immediately, subjects were required to move both hands from the initial center to target positions appointed by green and red blocks. The movement tasks must be completed before the 7th second. After the 7th second, both hands were required to move back to the initial center position. At the 11th second, one trial ended.

During the experiment, the gaze of subjects was asked to fix on the screen to avoid eye movement. The experiment was composed of four sessions, including the right-hand movement in the *R* or *L* direction with the left-hand movement in the *F* or *B* direction. One session consisted of five runs, and each run consisted of 16 trials. In total, we recorded 80 trials per session. It meant that, for each combination of directions, there were 80 trials uniformly. Between each run, the subjects were asked to perform a break of 2 min.

### Experimental Paradigm and Procedure

**Figure 2:**
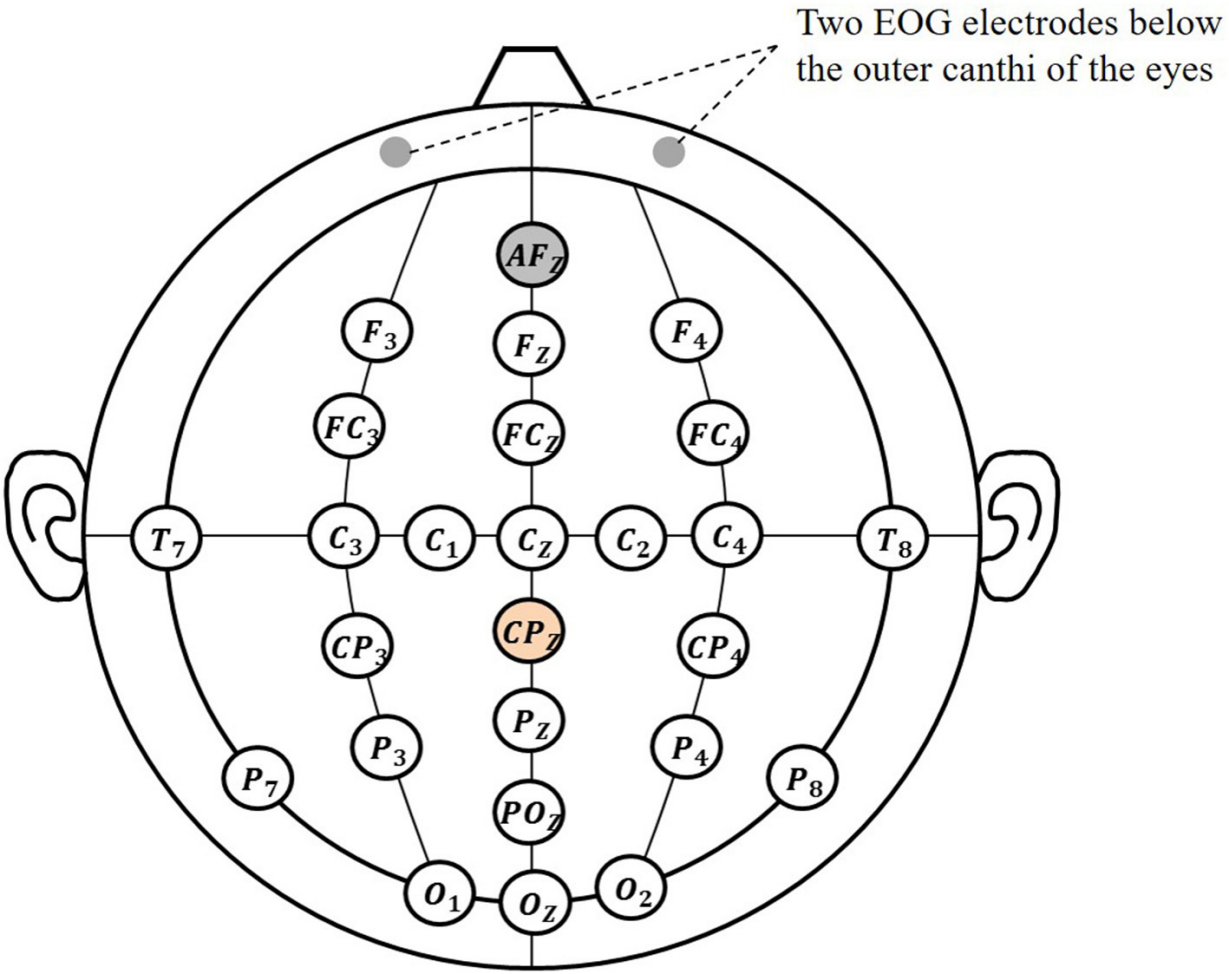
Used electrodes position diagram.

EEG signals were recorded by using a 64-electrode portable wireless EEG amplifier (NeuSen.W64, Neuracle, China), located at the following positions (according to the international 10–20 system): Cz, C1, C2, C3, C4, Fz, F3, F4, FCz, FC3, FC4, CP3, CP4, Oz, O1, O1, T7, T8, POz, Pz, P3, P4, P7, P8 (Wang et al., 2021), as shown in Figure 2. The selected electrodes involved the frontal, central, parietal, and occipital regions, which were related to the cognition, motion, perception function. The reference electrode was placed at CPz, and the ground electrode was placed at AFz. Electrooculogram (EOG) signals were recorded from two electrodes located below the outer canthi of the eyes. Two position-detecting sensors (FASTRACK) were positioned at tiger positions of both hands to track hands movement in real-time. The sampling rate of EEG signals was 1,000 Hz, and the sampling rate of position sensors was 60 Hz.

EEG, EOG, and position data were processed in MATLAB R2019b. The algorithm in steps for EEG signals-based primary hand movement direction decoding is listed in Table 1. For the signals preprocessing, EEG and EOG data were first down-sampled to 100 Hz, and each channel signal of EEG was re-referenced by subtracting the average of binaural electrodes. Baseline correction was used to eliminate the baseline drift for both EEG and EOG signals and common average reference was applied to remove common background noise for EEG signals. Eye movement artifacts were removed by using independent component analysis (ICA). The specific steps are as follows: (1) decomposing EEG signals into dependent component by applying independent component transform; (2) computing the correlation coefficients between the independent component and EOG signals; (3) rejecting the component whose correlation coefficient exceeds 0.4; (4) applying the inverse transformation on the remained component into EEG signals.

**Table 1:** EEG signals decoding algorithm steps.

**EEG signals decoding algorithm**
Step 1:	Down-sample EEG signals to 100 Hz and re-reference by binaural electrodes;
Step 2:	Baseline correction and common average reference for EEG signals;
Step 3:	Eye movement artifacts rejection by independent component analysis;
Step 4:	Band-pass filter in [0.01, 4] Hz by using fast Fourier transform filter;
Step 5:	Apply z-score for EEG signals normalization;
Step 6:	Extract features from EEG signals by using echo state network (ESN);
Step 7:	Reshape ESN features to one-dimension and use PCA for feature dimension reduction;
Step 8:	Apply LDA for primary hand movement directions decoding.

### Movement Related Cortical Potential (MRCP)

To correlate the neural activity during movement preparation and execution, MRCPs were extracted from EEG signals in the low-frequency band. After signal preprocessing, a zero-phase, 4th order Butterworth filter was used to filter EEG signals in the low-frequency band [0.01, 4] Hz. The weighted average filter was applied for electrode Cz to remove the spatial common background noise (Liu et al., 2018). To observe the difference of MRCPs for right-hand movement direction decoding, the MRCPs were obtained under condition of W-OHM for right-hand movement in *L* and *R* directions. The MRCPs under condition of W-OHM were average across all subjects.

### Feature Extraction

After signal preprocessing, a fast Fourier transform (FFT) filter was used to filter EEG signals in the frequency band [0.01, 4] Hz (Wang et al., 2010). For the primary hand movement decoding, an echo state network (ESN) was used to extract non-linear dynamics of EEG signals as the classification feature (in short ESN feature) (Sun et al., 2019).

**Figure 3:**
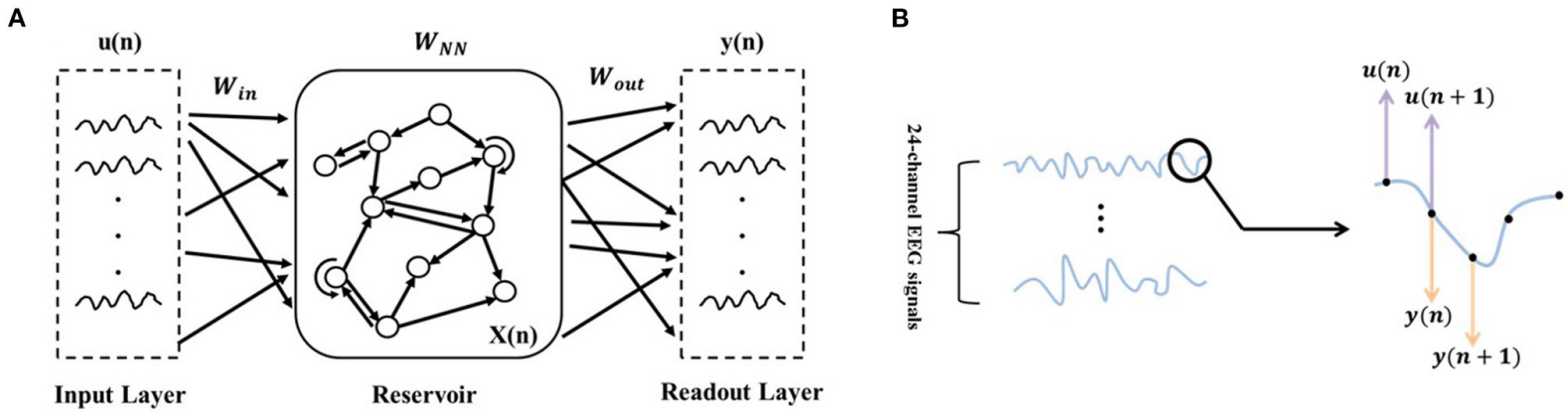
ESN schematic diagram. **(A)** Network structure diagram. **(B)** Illustration of the input and output data selection based on the time series of EEG signals. u(·) refers to the input data and y(·) refers to the output data.

As shown in Figure 3A, ESN is composed of the input layer, reservoir (hidden layer), and readout layer. The connecting matrix from the input to reservoir layers is defined as *W*_*in*_. The internal connection matrix of the reservoir is sparse, and is defined as *W*_*NN*_. Both *W*_*in*_ and *W*_*NN*_ are randomly initialized and kept invariant during network updating. The connecting matrix from the reservoir to readout layer is defined as *W*_*out*_, and it is updated with the input and output data. With *W*_*in*_, the ESN maps the input signals from the low-dimensional space into the high-dimensional non-linear space:


(1) $$\begin{array}{r} x\left(n\right)=f•\left({\;W}_{in}u\left(n\right)+{\;W}_{nn}x\left(n-1\right)\right), \end{array}$$

*f*• was set to be tanh• to realize the non-linearity of the network. In the high-dimensional non-linear space, the ESN model trains the *W*_*out*_ by linear regression (e.g. Ridge Regression).


(2) $$\begin{array}{r} W_{out}=Y_{t}X^{T}{\left(XX^{T}+\lambda_{r}I\right)}^{-1}, \end{array}$$

where λ_*r*_ is the readout regularization coefficient.

As the core part of the ESN, the reservoir layer has the following parameters: (1) sparsely connecting with the sparse degree *c*, (2) reservoir size (i.e., the number of neurons) *NN*, (3) spectral radius ρ (usually ρ < 1, to ensure that the effects of input and reservoir states on network vanishing after enough time), (4) the output of the reservoir layer at the current time *x*(*n*), and (5) internal connection matrix *W*_*NN*_ (randomly initialized and kept invariant during network updating). With the enormous and sparse reservoir layer, the ESN could capture the dynamics of a non-linear system. As mentioned in Waldert et al. (2008), EEG signals are non-steady and non-linear. From this perspective, we made a hypothesis that using the proposed method to establish the movement decoding model could obtain well-decoding performance.

In this paper, the multi-channel time-domain signals at the current time point were used as input signals, and the multi-channel time-domain signals at the next time point were used as output signals (as shown in Figure 3B). The output connection matrix *W*_*out*_, which could reflect the non-linear dynamics of EEG signals over time, was chosen as the ESN features for decoding. In addition to the parameters c and NN that have a major influence on the ESN performance and were determined in the subsequent training (by using mesh grid search), we empirically set the residual parameters: (1) ρ = 0.98; (2) *x*(0) was zero-matrix; (3) $\lambda_{r}=1\times 10^{-4}$. Besides, before encoding EEG signals to ESN features, z-score was applied for normalization, as follows


(3) $$\begin{array}{r} Z=\frac{X-\mu }{\sigma }, \end{array}$$

where *X* is the raw EEG signals before normalization, μ and σ are the mean and standard deviation of EEG signals, respectively. The original feature dimensions could be calculated by the following equation,


(4) $$\begin{array}{r} Num_{F}=C•\left(NN+C+1\right), \end{array}$$

where *Num*_*F*_ is the original feature dimension, *NN* is the reservoir size, *C* is the channel number. To suppress feature redundancy and accelerate computation, principal component analysis (PCA) was applied to reduce feature dimension. Choosing the principal component with the percentage above 99%, the dimension of ESN feature was reduced to 40.

### Classification

The fixed window [0, 1] s of the Go-Cue (i.e., [−0.5 0.5] s of the actual movement onset, calibrated by *FASTRACK*) was used for the primary hand movement direction decoding. Linear discriminant analysis (LDA) classifier was performed to decode the primary hand movement direction. The classification accuracy was used to measure the decoding performance, and the decoding accuracy was calculated by dividing the number of correctly classified test samples by the total number of the test samples. Mean classification accuracy was calculated by a 5 × 5 cross-validation. For the primary hand movement direction decoding under W-OHM, the classification accuracy was first calculated separately for the opposite hand movement in *F* or *B* direction and then averaged.

### Statistics

Power tables from Cohen were used to evaluate the number of participants needed to obtain a significant result (Puce et al., 2003). When 14 participants were involved in this experiment, partial eta squared (*R*^2^) was calculated as 0.417 by using ANOVA in IBM SPSS Statistics 25. Effect size for F-ratios was calculated as follows:


(5) $$\begin{array}{r} f^{2}=\frac{R^{2}}{1-R^{2}} \end{array}$$

When *f*^2^ is 0.7153, the equivalent effect size d is 1.6. At the given two-tailed α = 0.05 and the recommended power level of 80%, the number of participants needed for significant results was 9, which justified the sufficiency of subjects in our experiment.

## Results

### Neural Signatures

**Figure 4:**
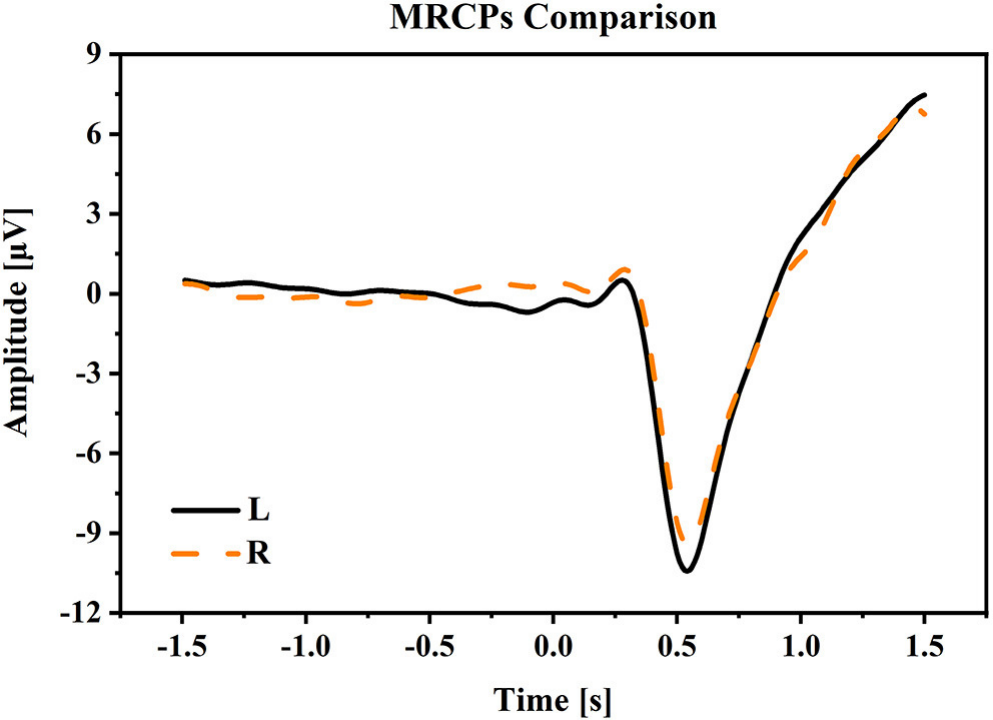
The averaged MRCPs at electrode Cz during the time period [−1.5, 1.5] s of Go-Cue. “L” and “R” refer to the right-hand movement in right and left directions. Note that time 0 s is the Go-Cue, and time 0.5 s is the actual movement onset.

**Figure 5:**
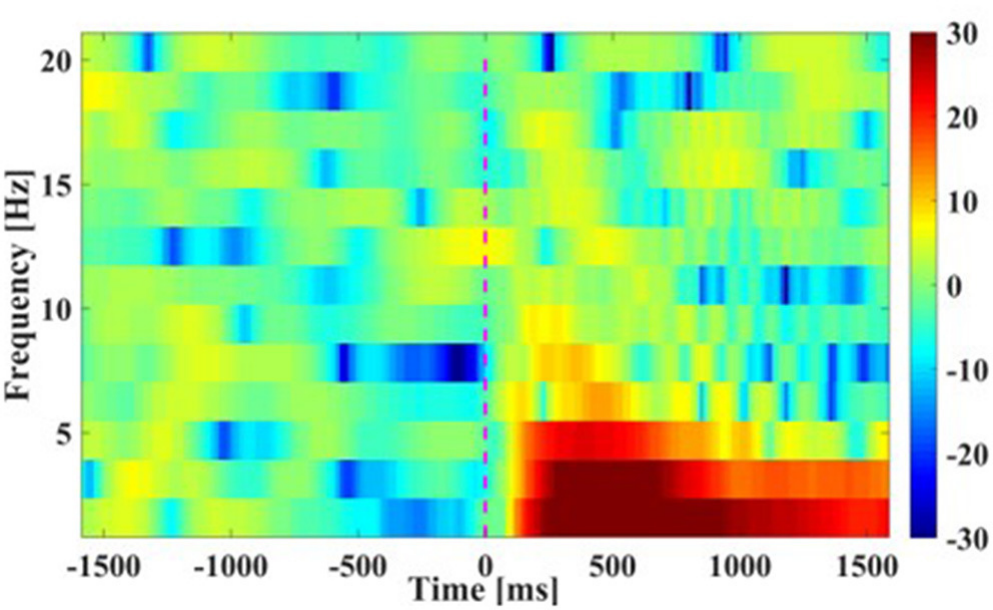
Time-Frequency plots of movement. The averaged results are in frequencies [0 16] Hz at Cz channel from −1.5 to 1.5 s of the movement cue onset. Note that time 0 s is the Go-Cue, which indicates the movement execution, and time 0.5 s is the actual movement onset.

**Figure 6:**
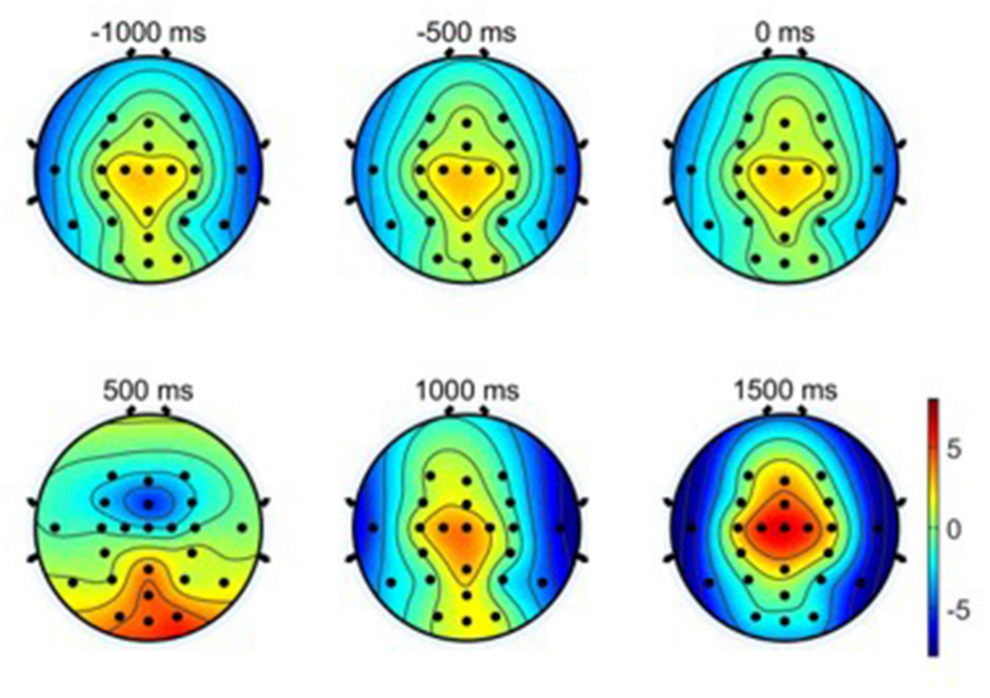
Averaged EEG potential topographic maps of hand movement across all subjects. Note that time 0 s is the Go-Cue, which indicates the movement execution, and time 0.5 s is the actual movement onset.

(1) Movement related cortical potential: Figure 4 shows the MRCPs at electrode Cz under the condition of W-OHM. Considering that the primary purpose of this study was to decode right-hand movement directions, the MRCPs associated with the right-hand movement in L and R directions were presented. The MRCPs were calculated from −1.5 to 1.5 s of the Go-Cue and averaged across all subjects. As shown in Figure 4, under all movement conditions, the amplitudes of MRCPs kept steady around 0 μV from −2 to 0 s, which was the movement preparation period. A positive peak was observed at around 300 ms, and after that a substantial negative shift arose and peaked at about 500 ms. The peak time of the negative shift was in agreement with the actual movement onset calibrated by FASTRACK (as labeled in Figure 1). For the movement under condition of W-OHM, the average negative shift maximums of the MRCPs for right-hand movements in R and L directions were −9.4153 and −10.4324 μV, respectively. By comparing the negative shift amplitudes of MRCPs between two primary hand movement directions, larger negative shift amplitude of the primary hand movement in L direction was found. However, this difference was not significant (Wilcoxon signed-rank test, *p* = 0.17). Furthermore, Wilcoxon signed-rank test showed that there was a significant difference between the MRCPs (from −1.5 to 1.5 s) associated with two primary hand movement directions (*p* < 0.01).

(2) Time-Frequency plots: Figure 5 presents the grand average time-frequency plots in the time period [−1.5, 1.5] s of the movement cue onset across all subjects. It was seen that a prominent increment in spectral power appeared after the movement cue onset in the low frequencies of smaller than 7 Hz (especially smaller than 4 Hz), indicating that main power modulations during bimanual movement was centralized in the low frequency band, and this result was similar to the finding in Robinson et al. (2015).

(3) Scalp topographical maps: The average EEG potential topographical distributions of the primary hand movements under the condition of W-OHM are shown in Figure 6. The scalp topographical maps were plotted from −1,000 to 1,500 ms with an interval of 500 ms in between. It was seen that cortical brain activities were steady from −1,000 to 0 ms with no specific modulation patterns. A significant decline in EEG potentials on central regions and a significant increment on occipital regions occurred from 0 to 500 ms. After 500 ms, peaks of these plots were centralized on central regions increasingly. Furthermore, the potential of EEG signals on temporal lobes turned into a negative shift and reached the negative maximum gradually, which was in line with the finding in Puce et al. (2003).

### Parameters Selection

The parameters of reservoir sparse degree c and reservoir size *NN* were critical to the performance of the proposed decoding model. The reservoir sparse degree is related to the number of neurons activated, and the reservoir size is associated with the complexity of the proposed model. Only with befitting parameters, the proposed model could capture the dynamics of EEG signals well. In this study, we used the mesh grid search to determine well-behaved subject-specific parameters *c* and *NN*. For determining the reservoir sparse degree *c*, the step size was set to be 0.1, and the search range was in [0.1, 0.9]. For determining the reservoir size *NN*, the search set was {10, 20, 30, 40, 50, 60, 70} (Sun et al., 2019).

**Figure 7:**
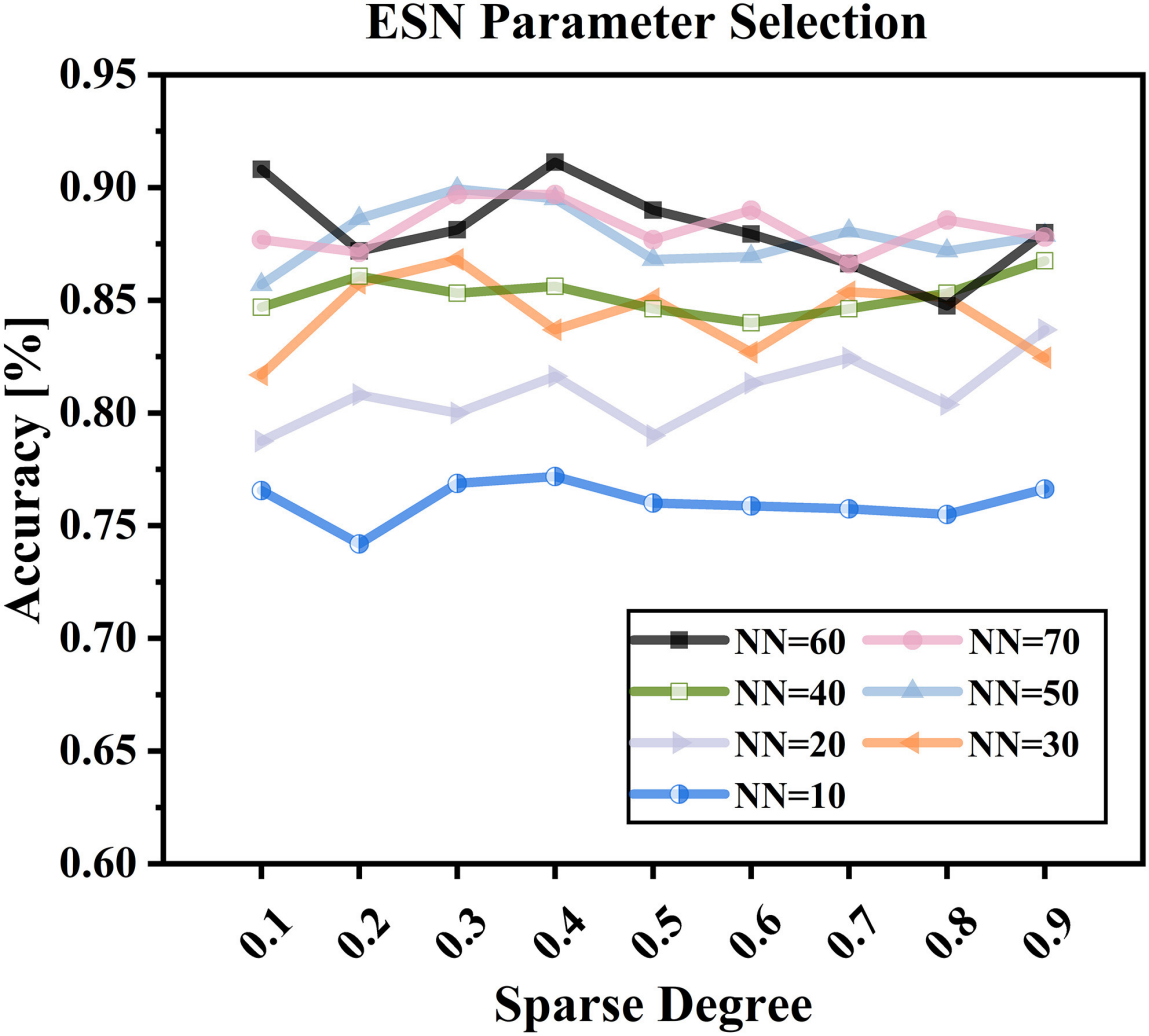
ESN parameter selection with different reservoir sparse degree *c* and reservoir size *NN*.

Table 2 shows the subject-specific decoding accuracies and parameters selected (*NN* and *c*) by using ESN-based models under condition of W-OHM. Figure 7 shows the example of the decoding accuracy of Subject 1 under condition of W-OHM against the reservoir sparse degree *c* and reservoir size *NN*. It was seen that, with the increase of the reservoir sparse degree *c*, the variation of decoding accuracy was slight for each reservoir size *NN*. Furthermore, with the increment of the reservoir size NN, the decoding accuracy was gradually improved and tended to be steady. The parameter combination with the best performance, i.e., “*c* 0.4, *NN* 60,” was selected for Subject 1 under condition of W-OHM.

**Table 2:** Subject-specific decoding accuracies and parameters selected (*NN* and *c*) under W-OHM by using the proposed model.

**Subject No**.	**acc [%]**	**NN**	**c**
S1	91.33	60	0.4
S2	92.44	70	0.7
S3	96.94	60	0.5
S4	91.81	50	0.1
S5	89.88	60	0.7
S6	97.69	70	0.3
S7	96.88	50	0.7
S8	86.56	70	0.3
S9	87.37	70	0.5
S10	82.75	60	0.4
S11	89.56	70	0.7
S12	84.25	70	0.6
S13	75.00	60	0.4
S14	90.19	70	0.9
Mean ± Std	89.48 ± 5.92	63.57 ± 7.18	0.51 ± 0.21

### Decoding Performance Comparison

**Figure 8:**
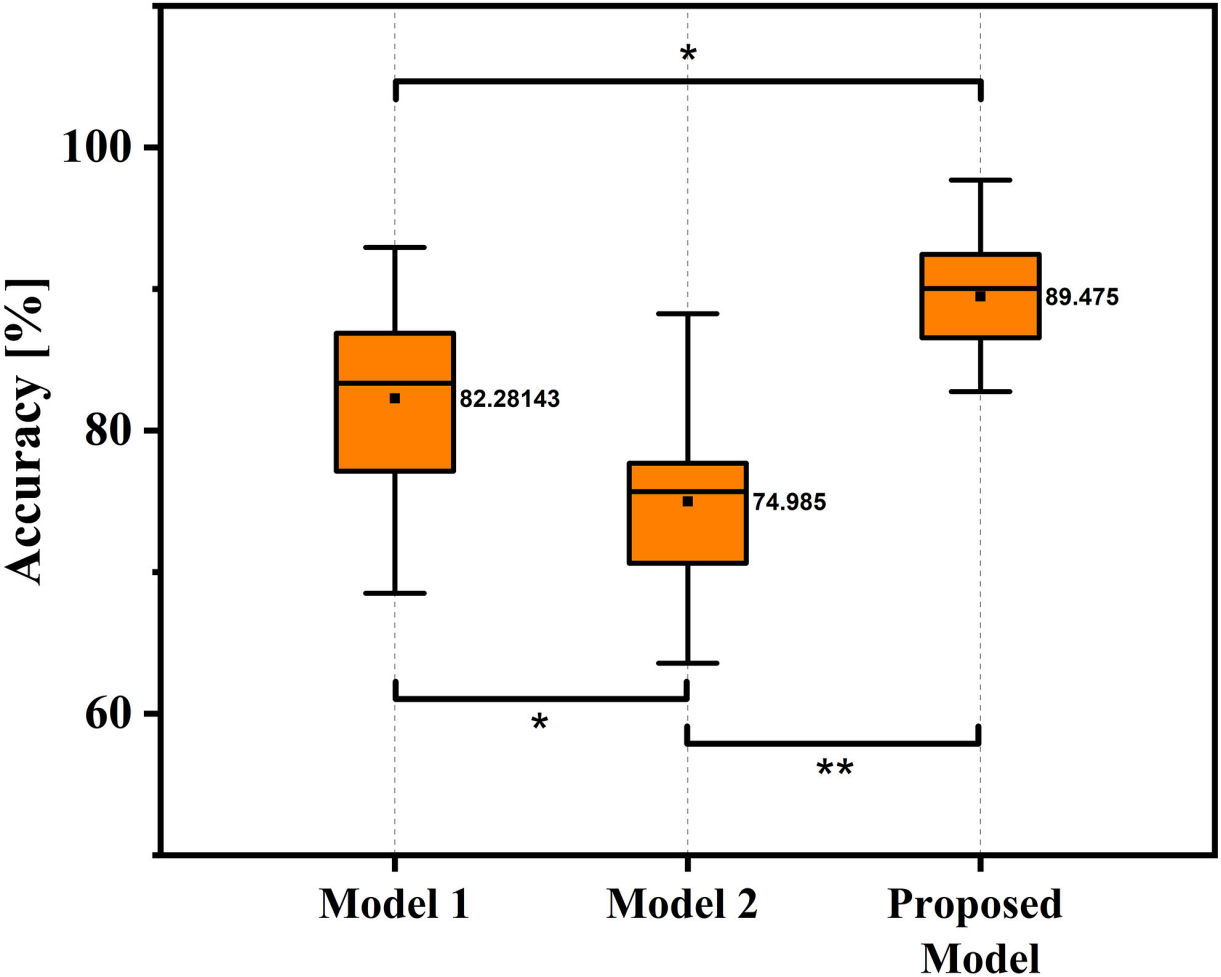
Box-Plots of average decoding accuracy under W-OHM by using three kinds of models. The asterisk marks significant differences. Tukey-Kramer *post hoc* test: *p < 0.05, **p < 0.01.

In this study, the classification performance of the proposed ESN model was compared with two models in (Wang et al., 2021), and we named two comparison models as Model 1 and Model 2 in this study. Specifically, Model 1 used potential amplitudes of EEG signals as feature and used LDA as classifier, and Model 2 used the sum of spectral power of EEG signals as feature and used LDA as classifier. For both model 1 and model 2, no personalized parameters tuning strategy was applicable. Table 3 shows the decoding accuracy comparison results based on different kinds of classification models under condition of W-OHM. Figure 8 shows the decoding accuracy comparison under condition of W-OHM among three kinds of models in the box-plot form. As shown in Table 3, the highest average decoding accuracy was obtained when using the proposed model, and was 89.48 ± 5.92%. Correspondingly, when using Model 1 and Model 2, the decoding accuracies were 82.28 ± 6.98% and 74.99 ± 6.13%, respectively. Significant differences were found between classification models by performing one-factor analysis of variance [*F*_(2, 39)_ = 16.88, *p* < 0.01]. The *post-hoc* pairwise comparison with the Tukey-Kramer method showed that there were significant differences between the Model 1 and Model 2 (*p* = 0.02), the Model 1 and the proposed model (*p* = 0.02), and the Model 2 and the proposed model (*p* < 0.01), as shown in Figure 8.

**Table 3:** Decoding accuracies across subjects under condition of W-OHM using different kinds of models.

**Subject No**.	**Model 1 [%]**	**Model 2 [%]**	**Proposed Model [%]**
S1	86.88	75.56	91.33
S2	81.25	77.69	92.44
S3	80.81	88.25	96.94
S4	71.56	70.19	91.81
S5	80.93	70.63	89.88
S6	89.25	77.10	97.69
S7	92.94	80.38	96.88
S8	74.56	70.81	86.56
S9	89.88	76.06	87.37
S10	77.13	73.31	82.75
S11	86.75	67.69	89.56
S12	86.06	82.75	84.25
S13	68.50	63.56	75.00
S14	85.44	75.81	90.19
Mean ± Std	82.28 ± 6.98	74.99 ± 6.13	89.48 ± 5.92

### Computational Time Comparison

Table 4 shows the computational time comparison results of different decoding models. The total computational time included the sum of signal processing, feature extraction, dimensionality reduction, and classification of a single sample. For the proposed model, averaged *NN* and *c* (64 and 0.5) calculated from Table 2 was used for calculating computational time. As shown in Table 4, the computational time of the Model 1, Model 2, and proposed decoding models was 13.1, 7.4, and 37.5 ms, respectively. It showed the feasibility of putting the proposed decoding model into real-time detection.

**Table 4:** Computational time of different decoding models.

**Computational Time [ms]**
**Model 1**	**Model 2**	**Proposed Model**
13.1	7.4	37.5

## Discussion and Conclusion

This paper explored using EEG signals to decode primary hand movement direction under the opposite hand movement. MRCPs, time-frequency plots, and scalp topographic maps were shown for neural signatures. The decoding model was built by using an ESN to extract non-linear dynamics parameters of MRCPs as decoding features. Experimental results showed that the proposed method performed well in decoding primary hand movement directions with the opposite hand movement. This paper is the first to investigate neural signatures and decoding of hand movement parameters under the opposite hand movement.

In this study, we followed the classic center-out paradigm (Robinson et al., 2013, 2015; Chouhan et al., 2018), and evolved it to both-hand center-out paradigm for the both-movement decoding. Specifically, we set the primary hand movement in L or R directions and the opposite hand movement in F or B directions. Considering that two hands often move in different directions in practical human-machine collaboration systems, we preliminarily set the primary hand and opposite hand movement in orthogonal directions. The advantages of this paradigm were that it was basic and representable for hand movement decoding and its experiment results were general and could be extended to practical hand movement decoding problem. By comparing the negative shift amplitudes of MRCPs between two primary hand movement directions, a larger negative shift amplitude of the primary hand movement in *L* direction was found (*L*: 10.4324 vs. *R*: 9.4153 μV). This result was in accord with our previous study, which indicated that the larger negative shift amplitude of MRCP might be related to the higher torque-level for the leftward motion of the right arm (Wang et al., 2021). The increment of spectrum energy was mainly centralized in the low-frequency band, which was similar to the finding in Waldert et al. (2008), which indicated that hand movement directions could be decoded from power modulations in the low-frequency band. The increment of event-related potentials (ERPs) on central regions and the decrement of ERPs on temporal regions were found in scalp topographic maps from 500 to 1,500 ms, which was in line with the findings in Puce et al. (2003) and (Wang et al., 2021), respectively.

Experimental results showed that the proposed decoding model outperformed the models used in (Wang et al., 2021 (89.48% vs. 82.28% or 74.99%). One main reason for the results is likely that the proposed method could capture more discriminable information of MRCPs for decoding hand movement direction. This ability of the proposed model may be because ESN can establish a complex non-linear dynamic system of EEG signals with a large reservoir size and complex transmission relationships between neurons and can constantly update the network parameters according to the information from the previous moment. Furthermore, compared with other neural network (e.g., convolution neural network and deep belief network), ESN, as one kind of recurrent neural network, could capture the nonstationary and nonlinear features and is good at dealing with the time sequence problem.

This work has values in at least two implications. First, the proposed method can capture more meaningful non-linear information of MRCPs for decoding hand movement direction. Thus, this work may open a new avenue to decode other hand movement parameters, such as velocity and trajectory. Second, since, for human augmentation, many tasks need to be carried out by the movement of both hands, these findings can lay a foundation for the future development and use of human augmentation systems based on hand movement decoding from EEG signals.

However, at least three limits exist in this work. First, although the proposed decoding method of primary hand movement direction under the opposite hand movement performed well, the movement intensity of the left hand was kept at a certain level. For further exploration of the decoding of primary hand movement under the opposite hand movement, different kinds and intensities of the opposite hand movement, including more natural and complex movement, should be considered. Second, like many studies in the field of using EEG signals to decode hand movement (Robinson et al., 2015; Chouhan et al., 2018; Schwarz et al., 2020), we used able-bodied subjects to investigate neural signatures and decoding of hand movement direction. However, it is unclear whether these results can be extended to persons with disabilities. Thus, more subjects, especially the target users (including the disabled), should be applied to validate these findings further. Third, in this study, all recruited participants were right-handed, and 1 female among which was recruited. Though the influence of handedness and gender were focused on in this paper, handedness and gender may be the factors that influenced the decoding of primary hand movement under opposite hand movement, which could be explored in future.

Our future work will be dedicated to solving the weaknesses mentioned above, including using more types of hand movement directions given more types and intensities of the opposite hand movements, using more subjects and even some persons with motion impairment and exploring the influence of handedness and gender on decoding.

## Data Availability Statement

The raw data supporting the conclusions of this article will be made available by the authors, without undue reservation.

## Ethics Statement

The studies involving human participants were reviewed and approved by the Beijing Institute of Technology Research Ethics Committee. The patients/participants provided their written informed consent to participate in this study.

## Author Contributions

In particular, JW, LB, and WF contributed to the design of this work. JW: methodology, validation, formal analysis, and writing—original draft. LB: conceptualization, resources, writing—review and editing, and funding acquisition. WF: software and data curation. All authors contributed to the article and approved the submitted version.

## Funding

This work was supported in part by National Natural Science Foundation of China under Grant (No. 51975052).

## Conflict of Interest

The authors declare that the research was conducted in the absence of any commercial or financial relationships that could be construed as a potential conflict of interest.

## Publisher's Note

All claims expressed in this article are solely those of the authors and do not necessarily represent those of their affiliated organizations, or those of the publisher, the editors and the reviewers. Any product that may be evaluated in this article, or claim that may be made by its manufacturer, is not guaranteed or endorsed by the publisher.
